# A Critical Time Window for Organismal Interactions in a Pelagic Ecosystem

**DOI:** 10.1371/journal.pone.0097763

**Published:** 2014-05-20

**Authors:** Kelly J. Benoit-Bird, Margaret A. McManus

**Affiliations:** 1 College of Oceanic and Atmospheric Sciences, Oregon State University, Corvallis, Oregon, United States of America; 2 Department of Oceanography, University of Hawaii Manoa, Honolulu, Hawaii, USA; University of Connecticut, United States of America

## Abstract

To measure organismal coherence in a pelagic ecosystem, we used moored sensors to describe the vertical dynamics of each step in the food chain in shelf waters off the west shore of Oahu, Hawaii. Horizontally extensive, intense aggregations of phytoplankton, zooplankton, and micronekton exhibited strong diel patterns in abundance and vertical distribution, resulting in a highly variable potential for interaction amongst trophic levels. Only around dusk did zooplankton layers overlap with phytoplankton layers. Shortly after sunset, micronekton ascended from the deep, aggregating on the island's shelf. Short-lived departures in migration patterns were detected in depth, vertical distribution, density, and total abundance of micronekton when zooplankton layers were present with typical patterns resuming within one hour. Layers of zooplankton began to disappear within 20 minutes of the arrival of micronekton with no layers present after 50 minutes. The effects of zooplankton layers cascaded even further up the food chain, affecting many behaviors of dolphins observed at dusk including their depth, group size, and inter-individual spacing. As a result of these changes in behavior, during a 30-minute window just after dusk, the number of feeding events observed for each dolphin and consequently the feeding time for each individual more than doubled when zooplankton layers were present. Dusk is a critical period for interactions amongst species in this system from phytoplankton to top predators. Our observations that short time windows can drive the structure and function of a complex suite of organisms highlight the importance of explicitly adding a temporal dimension at a scale relevant to individual organisms to our descriptions of heterogeneity in ocean ecosystems.

## Introduction

Interactions between predators and prey have shaped all life on earth, affecting individual behavior, species morphology, population dynamics, species diversity, community structure, and ecosystem function [1]. The probability of these interactions between trophic levels is largely a function of the density of predator and prey and the overlap of their populations. [2] The roles of ecological process have been shown to be scaled by the spatial context in which an organism is found [3]. In pelagic systems, there are four dimensions of overlap to consider – the x-y or horizontal plane, depth (z), and time (t). Much attention has been paid to the horizontal spatial relationships between predator and prey and their dependence on scale [4], [5], [6], [7]. However, the challenge of accessing organisms beneath the surface of the ocean has made adding measurements of vertical distribution of both predator and prey simultaneously difficult [8], [9], [10].

The emphasis on spatial distribution in ecological interactions (and the development of the field of landscape ecology) was, in part, driven by seminal experimental and theoretical work in systems with a two-dimensional surface onto which one member of the dyad is tied physically [11], [12], [13] or computationally [14]. However, even a description of spatial coherence in three dimensions is not sufficient to understand the coincidence of organisms. The fourth, temporal dimension is particularly crucial in pelagic systems because even immobile organisms can be moved by currents and, unlike terrestrially influenced three-dimensional habitats [15], [16], there are relatively few fixed features to provide cover or anchor behavior. Surveys of predator and prey densities implicitly assume a constant relationship within each observation, making it possible to observe correlations at climatological [17], [18], annual [19], seasonal [20], [21], or even daily [22] time scales. Knowledge of the behavioral state of observed predators can allow correlations at smaller time scales, sometimes even approaching a single foraging event [8], [10], [23], [24]. However, it is typically difficult to connect the small scale observations of an individual predator to the larger scale observations of populations or communities necessary to understand the ecology of the system [25].

The complexities of resource heterogeneity in both space and time are critically important to ecosystems; they may underlie the timing of outbreaks and blooms, successional patterns, stability of predator-prey interactions, and coexistence of multiple competitors for the same resources [26]. However, adding temporal dynamics to our understanding of spatial variance has been identified as the most challenging task in ecosystem description and prediction [27]. To move beyond interpretation of temporally separated snapshots of a system, here, we use high-resolution moored sensors to describe the vertical dynamics of each component of Hawaii's nearshore pelagic ecosystem, a relatively well-described food web beginning with phytoplankton and culminating in spinner dolphins (*Stenella longirostris*). The distribution of biomass in each of these trophic levels is spatially heterogeneous, which we showed in previous analyses affects predator-prey interactions throughout the system [28]. In describing the importance of spatial pattern, however, our previous efforts did not measure true coherence between predators and prey as they focused on long-term temporal dynamics. By not addressing vertical position of aggregations of organisms in the water column or time variance at scales of less than a day, the mechanisms of these interactions remain obscured. Particularly as short time scales and vertical position are predicted to be important based on the strong diel patterns in both local biomass and vertical distribution observed in each step of the food chain (spinner dolphins [23]; zooplankton [29]; phytoplankton [30]; micronekton [31]). In this contribution, we use fine vertical (tens of cms) and temporal (one sample every second to every 15 minutes, depending on the instrument) resolution descriptions of the distribution of biomass in the food web to elucidate the interactions between predators and prey in both space and time.

## Methods

**Figure 1:**
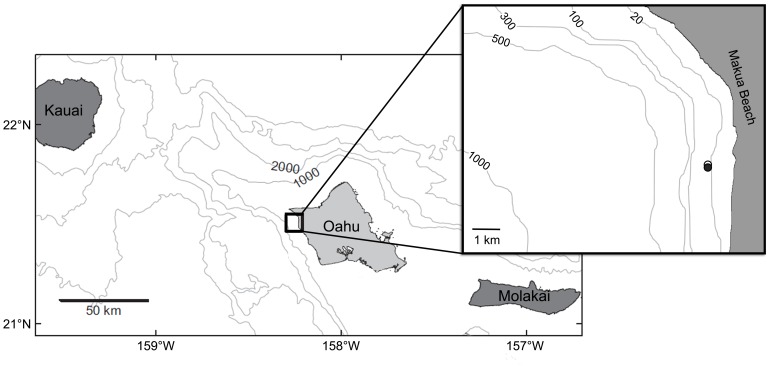
A map of the field site off leeward Oahu, Hawaii showing the location of the ship-based sampling (open circle) and the moored sensors located approximately 100 m away (filled circle), both on the 25 m isobaths.

Over two, three-week periods in the spring of 2009 and 2010, we used moored sensors complemented by shipboard surveys to measure the physical and biological properties of the shelf off the leeward coast of Oahu, Hawaii. This section of coastline runs north to south with little variation, and isobaths are roughly parallel to the shoreline. The study was conducted near the 25 m isobath from 20 April to 12 May 2009 and 10 April to 5 May 2010 in the area of 21° 30.5 N, 158° 14.2 W (Figure 1), roughly 1 km from the shoreline and 5 km inshore of the habitat that serves as the daytime habitat for mesopelagic animals of the deep-scattering layer [31].

### Ethics Statement

Observations of spinner dolphins were conducted under a US National Marine Fisheries Service Permit 1000–1617. The area accessed was not privately owned or protected and no protected species were sampled. This effort complied with all relevant regulations.

### Moorings

A moored autonomous profiler (the Seahorse; Brooke Ocean Technology) collected temperature, salinity, pressure (SeaBird SBE-19 CTD), dissolved oxygen (SBE-43), and chlorophyll fluorescence (WET Labs ECO-FLS) data every 30 minutes between the near-bottom and the near-surface with <1 cm vertical resolution.

Throughout each study period, an upward looking multi-frequency echosounder (ASL Environmental Acoustic Water Column Profiler: 200, 420, 740 kHz) collected acoustic backscatter data from zooplankton, micronekton, and dolphins once per second. The echosounder used a pulse length of 256 ms, had a 3-dB beamwidth of 7 degrees, and a vertical resolution of 1 cm. The echosounder was calibrated in two ways: in a seawater tank using a reference sphere [32] and at sea using both a reference target as well as a comparative approach with the calibrated shipboard echosounders.

### Shipboard sampling

Ship-based sampling was conducted from the 9 m *Alyce C*. anchored near the moored sensors. Sampling of zooplankton, micronekton and dolphins with echosounders and phytoplankton, zooplankton, and micronekton with a high-resolution profiling package was carried out continuously for 24 hours during the full, new, and quarter moon phases in each year. Additional sampling conducted over eight shorter intervals in each year that were dispersed over each study period. These efforts were supplemented by periodic zooplankton net tows and phytoplankton bottle samples.

### Echosounders

Downward-looking echosounders mounted 1 m below the surface (calibrated [32] split-beam Simrad EK 60 s at 38 kHz (12° beamwidth) and 70, 120, and 200 kHz (7° beamwidth) and Simrad ES60, 5° single beam at 710 kHz, each with an outgoing pulse length of 256 µs and a pulse rate of 8–10 Hz) were used to continuously measure the distribution and density of micronekton and zooplankton aggregations. A 200 kHz multibeam sonar (Kongsberg-Mesotech SM2000) was also deployed on the rigid pole. This system has a 120 degree by 1 degree field of view that was used to observe groups of foraging dolphins [23].

### Profiler

The physical and biological characteristics of the water column were measured near-continuously with a profiling package during each sampling period. Profiles covered 1 m from the surface to 1 m above the bottom approximately every 4 minutes throughout each sampling period. To maximize the vertical resolution of the profiles the instrument package was ballasted to achieve an average descent rate of 10 cm s^−1^ and was decoupled from ship motion during descent by allowing cables to remain slack. The profiling package was equipped with a CTD (SeaBird 25; temperature, salinity, pressure), a dissolved oxygen sensor (SBE-43), and a fluorometer to measure chlorophyll a fluorescence (WETLabs WETStar). Data from CTD casts were low passed filtered and edited for loops before the raw variables were converted to variables of interest using factory calibrations.

The profiling package also had a Tracor acoustic profiling system (TAPS) which uses acoustical scattering at six frequencies (265, 420, 700, 1100, 1850 and 3000 kHz) to quantitatively estimate zooplankton abundance in size classes [33]. Volume scattering strength profiles were averaged into 0.2 m vertical bins and transformed to estimates of zooplankton biovolume in equivalent spherical diameter (ESD) classes via a constrained, non-linear, least-squares algorithm [34], [35], [36], that employed a simple spherical model; a choice guided by the body forms observed in net tows. These estimates of biovolume were converted to estimates of density by dividing the biovolume at a given size by the volume of a spherical animal of that ESD. Large individual targets (e.g. micronekton) were rarely sampled due to the small sample volume of TAPS (about 3 l). After initial processing, TAPS profiles were analyzed for the presence of discrete layers, as well as vertically integrated to provide an estimate of total zooplankton abundance.

To obtain information on the taxonomic composition, numerical density, and size of micronekton, the profiler was equipped with a two-camera low-light video system with infrared illumination. Still views were extracted every 0.25 m from each of the two cameras and analyzed following Benoit-Bird and Au [31].

### Net tows

Periodically throughout shipboard sampling efforts, vertical net tows were conducted with a 0.5 m opening/closing, 200 µm mesh net equipped with a pressure sensor with real time communication (Simrad PI32) and flow meter modified to spin only on the upcast (General Oceanics). These tows were used to provide information on the identity and density of zooplankton in identified features and more generally throughout the water column. When an acoustic feature of interest was identified during shipboard sampling, the feature was discretely sampled at least once. Each targeted samples was followed by a sample integrated from 1 m above the bottom to the surface. Water-column integrated samples were also taken at regular intervals when no features of interest were identified. Samples were preserved in a buffered 5% formalin solution in seawater for later analysis following the methods described in Benoit-Bird et al.[29].

## Data Analysis

### Echosounder data

All echosounder data were analyzed using Myriax's Echoview software. For shipboard echosounders, all echoes from solitary individual targets, that is targets at densities lower than one per sampling volume were identified as large individual fish or marine mammals and removed from the data. In moored data, intense, large targets that were present at all frequencies were identified as marine mammals [23] and removed from the data set. The remaining volume scattering data from both the shipboard and moored sensors were thresholded at a value of −80 dB and integrated in 10 second horizontal by 0.30 m vertical bins. The volume scattering in each bin was then compared across all frequencies. Those bins that had volume scattering that did not increase by more than 2 dB with any increasing step in frequency were classified as micronektonic animals [37]. Of the remaining bins, those that had the scattering at least 4 dB higher at the two highest frequencies used than the lowest were classified as zooplankton [38], [39]. These two classifications accounted for 95% of all intervals for both echosounder systems.

Using only data classified as zooplankton, the total scattering at 200 and 710 kHz for the shipboard echosounders and 420 and 740 kHz for the moored echosounder were integrated over the entire water column in 10 s bins. Using only data classified as micronekton, raw 200 kHz volume scattering from both the moored and shipboard echosounders was integrated into 10 second by 0.5 m bins. Numerical density of micronektonic animals was calculated from the 200 kHz volume scattering data, determined to be the most accurate frequency for this estimate [37], using echo energy integration. Estimates of individual target strength were made by combining laboratory target strength measurements with animal size and identity information from the video system on the high-resolution profiler averaged across all recordings made at the same time of day and depth as the echosoundings.

### Identification of biotic layers

Both fluorescence and acoustic scattering were found in discrete, persistent features or layers. Layers in all data sets were identified following the general approach detailed in Benoit-Bird et al. [40] and summarized here. Layers in scattering consistent with micronekton were identified in the 200 kHz data from both moored and shipboard echosounders, while layers in scattering consistent with zooplankton were analyzed at all frequencies 200 kHz and above. A running, 5 m vertical median was taken for each profile which was an individual cast of TAPS or the fluorometer or 10 s averaged classified echosounder data (micronekton or zooplankton, analyzed separately) to define the local background in acoustic scattering or fluorescence. If the maximum measurement exceeded this background by 1.25 times for at least two consecutive casts or 30 s of echosounder data, the feature was considered a layer. The points at which scattering crossed below the running median were used to define the upper and lower edges of the layer. Scattering or fluorescence between these two points was integrated for each layer. Layer peak depth was defined as the point at which the layer reached a maximum value while median depth was the depth at which the integrated value within the layer was split in half. For plankton layers, thickness was calculated as the range of values within half the peak intensity of the layer, sometimes called the full width half maximum (FWHM), for comparison with previous studies. The average of these characteristics was calculated for each detected layer from the mooring data for statistical comparisons. The persistence of all zooplankton layers less than 5 m in thickness was characterized from the moored echosounder as the total time a layer was identified with no gaps in detection greater than 10 seconds. This analysis was not conducted on the time series of fluorescence data because of its low temporal resolution of one profile every 15 minutes.

### Zooplankton effects on micronekton and dolphins

To test for potential effects on micronekton occurring before their arrival at the field site, an ANOVA was used to examine the effects of zooplankton layer presence on the timing of the arrival of micronekton at the site. To determine if the vertical distribution of the mesopelagic layer was affected by the presence of a zooplankton layer, the median depth, thickness, integrated abundance, and mean density of the layer of mesopelagic animals was measured on each night 30 min after sunset as well as 2 hours after sunset as reported by the US Naval Observatory. A multiple Analysis of Variance (MANOVA) was used to test for the effects of zooplankton layer presence on micronekton layer characteristics.

An ANOVA was used to determine the effect of zooplankton layers on spinner dolphin median depth measured 15–45 min after sunset. To examine other effects of zooplankton on spinner dolphin behavior. we examined data from 18 days of shipboard data that covered at least the 30 minutes before sunset and two hours after. On 11 of these days a zooplankton layer was present. Following the methods of Benoit-Bird and Au [23], we used the multibeam sonar system to count the number of dolphins within each foraging group, the average spacing between pairs of dolphins within each foraging group, and the average time each pair spent inside the circle of foraging dolphins, a measure of feeding time for all groups observed 15–45 minutes and 90–120 minutes after sunset. We used a MANOVA with sampling date as a covariate to determine if there was a significant effect of zooplankton layer presence on these measures of spinner dolphin behavior in each time interval. We counted the number of dolphin groups observed during each of these 30 minute intervals and used an ANOVA to determine if there was an effect of zooplankton layer presence on the number of dolphin groups observed.

### Micronekton effects on zooplankton layers

To examine the impact of micronekton on zooplankton layers, scattering at 740 kHz from the moored echosounder was integrated over the thickness of detected zooplankton layers. Scattering within a layer was tracked in 5 minute intervals from 15 minutes before the first detection of micronektonic animals to 60 minutes after. The average value for each layer in the three, 5 minute intervals immediately before the arrival of micronekton was subtracted from each subsequent observation of the same feature to normalize the results.

## Results

### Fluorescent layers

A total of 206 discrete, fluorescent layers were identified from the moored data set. Of these, all but 5 of these were thinner than 5 m. A comparison of layers detected with the echosounder mooring and those detected with the profiler showed detection rates and vertical locations coincided. Fluorescence within these layers accounted for 18%–94% of the water column integrated fluorescence while layers covered 1–20% of the water column with the average thickness layer covering about 10% of the water column's depth. The fluorescence from phytoplankton layers was highly disproportionate to their vertical extent.

**Figure 2:**
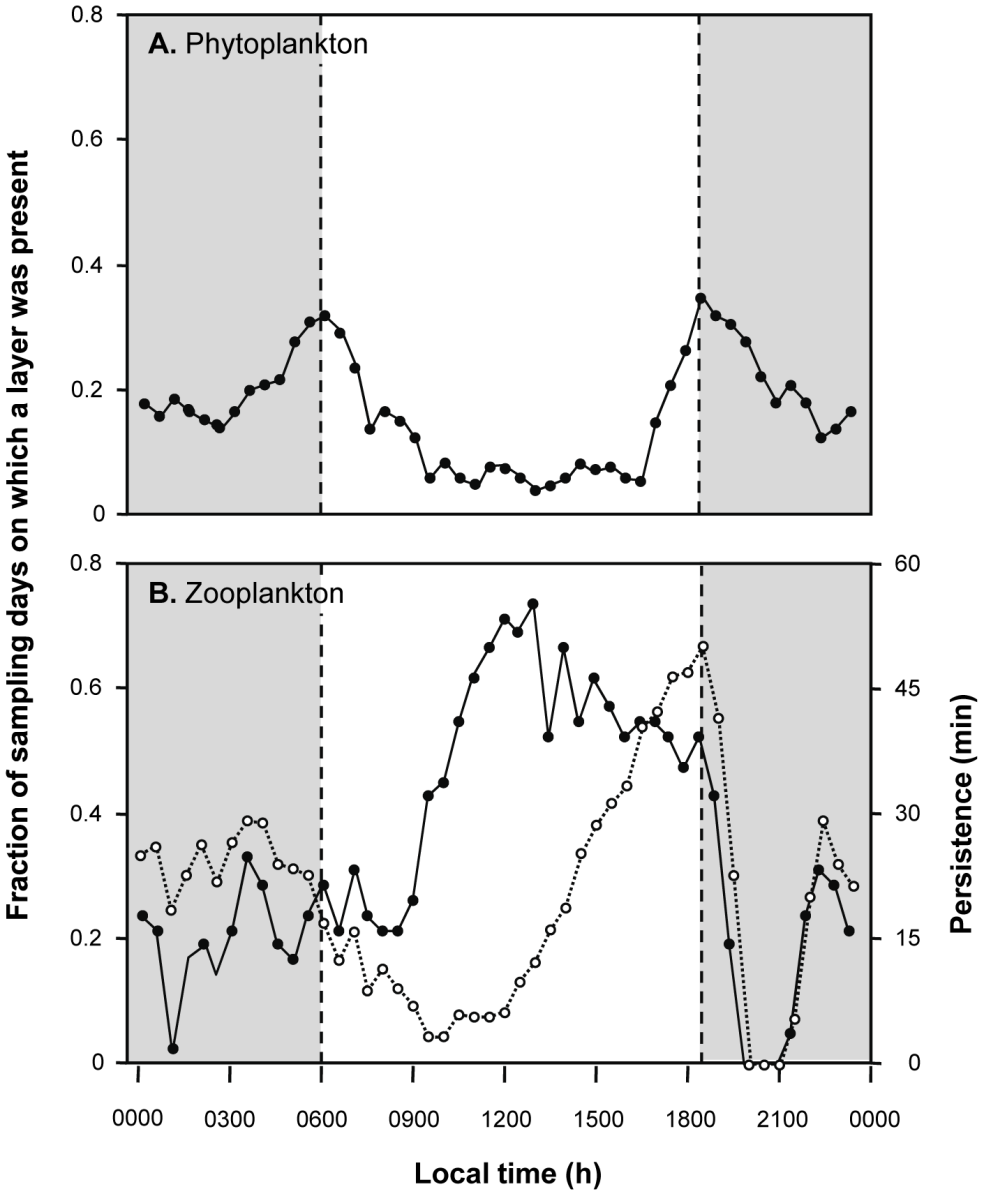
The frequency of occurrence of layers of phytoplankton (A) and zooplankton (B) as a function of local time detected using moored instruments across the two, 3-week studies is shown in the black symbols and solid lines. The y-axis shows the fraction of the 42 sampling days on which layers were detected in each time interval. Dusk and dawn are indicated by dashed lines separating the gray (night) from white (day) regions. Open circles and dashed lines in B indicate the average persistence, the total time a zooplankton layer was identified with no gaps in detection greater than 10 seconds, of zooplankton layers.

**Figure 3:**
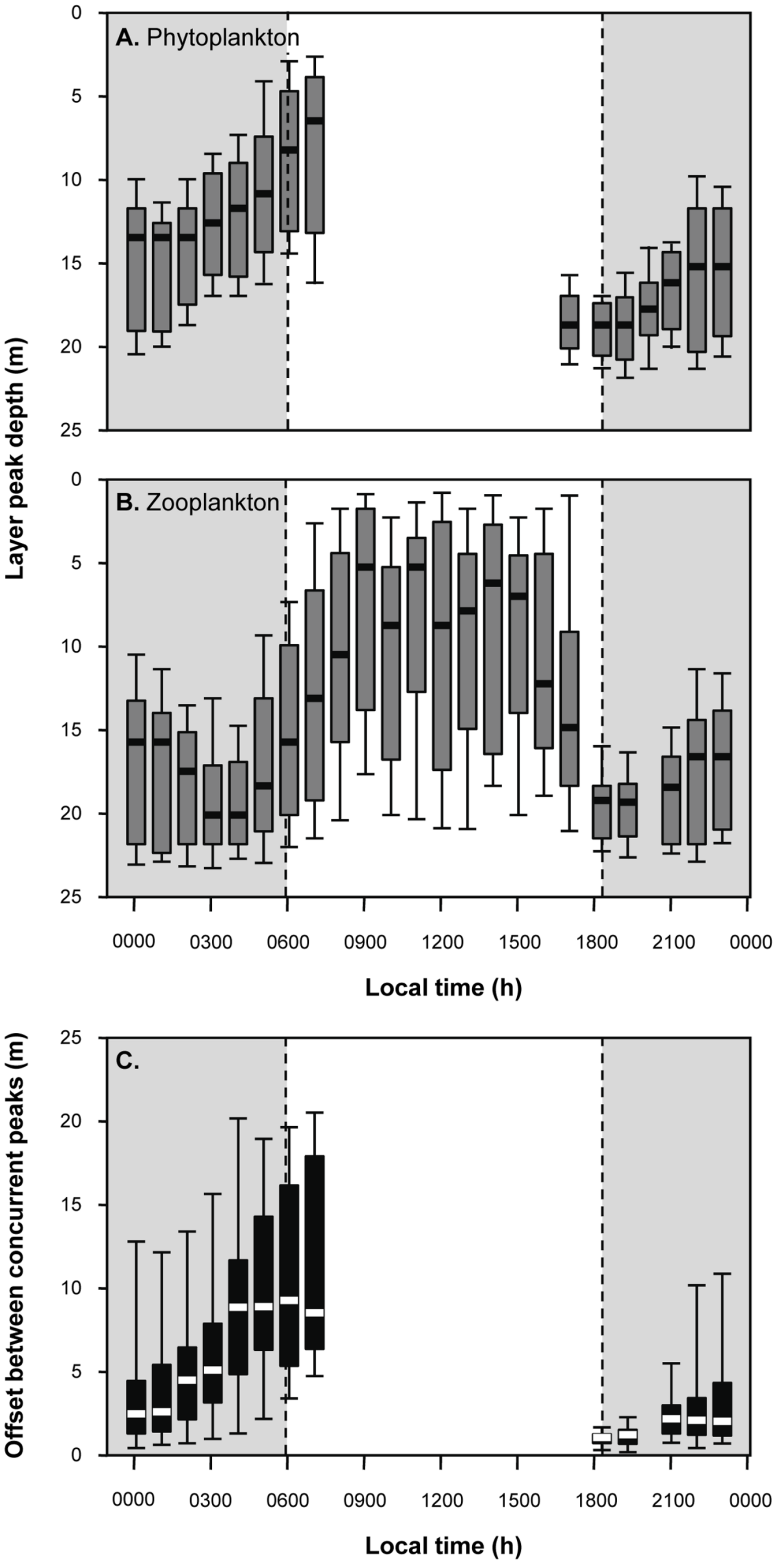
The peak depth of phytoplankton (A) and zooplankton layers (B) as well as the vertical offset between concurrently detected layer peaks (C) is shown as a function of local time. In each plot, heavy bars indicate the median of the distribution, the box one interquartile range, and the error bars show the 95% confidence interval.

Fluorescent layers showed a diel pattern during both sampling years (Figure 2A), with more layers found at night than during the day and peaks in layer frequency at dawn and dusk. The depth of layers also showed a diel pattern, with the shallowest layers early in the morning and the deeper layers around dusk (Figure 3A). Note that too few layers were present between 0800 and 1600 to represent their vertical distribution with any confidence. Although we profiled continuously with the high-resolution shipboard profiler during the surveys, we only discuss layers occurring between 1600 hours and 0800 hours. During these evening and early morning hours, the average PAR in the upper 5 m of the water column was extremely low (<200 µE m^−2^ s^−1^); thus, we avoid non-photochemical quenching of fluorescence. For comparison, the maximum PAR was 1,469 µE m^−2^ s^−1^ at 1230 hours on 3 May.

### Zooplankton acoustic layers

A total of 372 layers with scattering characteristics consistent with zooplankton were identified from the moored data set. Of these, 361 (all but 11) were thinner than 5 m and only 4 identified layers were between 3 and 5 m in thickness. A comparison of layers detected by the moored echosounder and those detected by TAPS showed that detection rates and vertical locations were identical. Similarly, the same layers were detected using the 710 kHz shipboard echosounder and the 740 kHz moored echosounder. Zooplankton layers during daylight hours were detectable at all frequencies used by the echosounder mooring and all frequencies 120 kHz and above on the shipboard echosounders, however, at night when mesopelagic animals were abundant, zooplankton layers were not detectable at frequencies 200 kHz and below.

A criterion of 1.25 times the local background was used to define layers, however, in zooplankton layers detected in mooring data, the minimum intensity of scattering at 740 kHz was 2.65 times the local background, the maximum was approximately 8000 times the local background, and the average layer was about 1000 times as intense the local background. Acoustic scattering at 740 kHz from these layers accounted for between 31% and 100% of the scattering attributable to zooplankton in the water column while layers covered between 1 and 20% of the water column with the average thickness layer covering about 7% of the water column. These layers of zooplankton were intense and scattering from layers of zooplankton was highly disproportionate to their vertical extent.

The fraction of sampling days in which zooplankton layers were detected showed a diel pattern during both sampling years (Figure 2B), with more zooplankton layers detected during the day than at night. No layers of zooplankton were ever detected between 90 minutes and 3 hours after sunset. The persistence time of zooplankton layers also showed a diel pattern (Figure 2B) with layers increasing in persistence time throughout the day until they peak at dusk before disappearing entirely. Figure 3B shows how the depth of zooplankton layers changed throughout the day with layers generally found shallower during the day than at night. The depth distribution of layers during the day also had a much larger spread. Layer position was more consistent at night with the narrowest range of depth values found around dusk.

**Figure 4:**
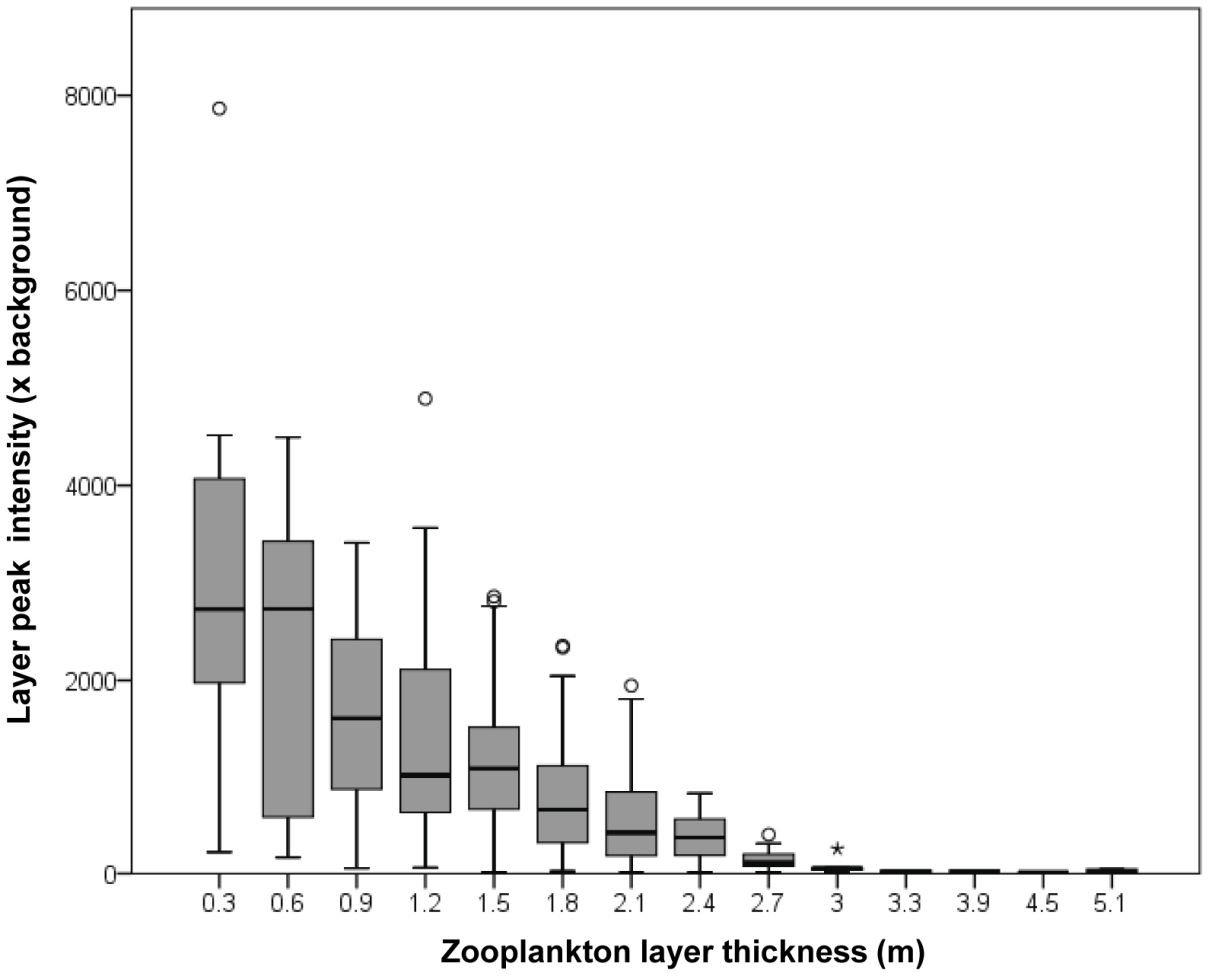
The peak intensity of layers detected at 740 kHz from the moored sensor is shown as a function of layer thickness. Heavy bars indicate the median of each distribution, the box one interquartile range, the error bars the 95% confidence interval, open circles outliers, and stars are those values that are least three box lengths from the median. All extreme outliers were mode 1 layers, those that had much stronger volume scattering at 420 than 740 kHz, revealed by net tows to be comprised of hyperrid amphipods rather than the copepods that made up the majority of layers.

Zooplankton layers detected in mooring data showed a significant pattern in peak intensity relative to the local background as a function of layer thickness (Figure 4), which can be described by an exponential curve (Peak intensity  = 3.93.6 e^−1.17 * thickness^; R^2^ = 0.38; p<0.01). There was no significant relationship between layer thickness and the total integrated scattering within the layer at 720 kHz, even when outliers and thicknesses with small sample sizes were removed (R^2^ = 0.02, p>>0.05). The median integrated scattering within each layer was about 250 m^2^nmi^−2^ at 740 kHz, regardless of layer thickness.

### Identification of zooplankton layer constituents

**Figure 5:**
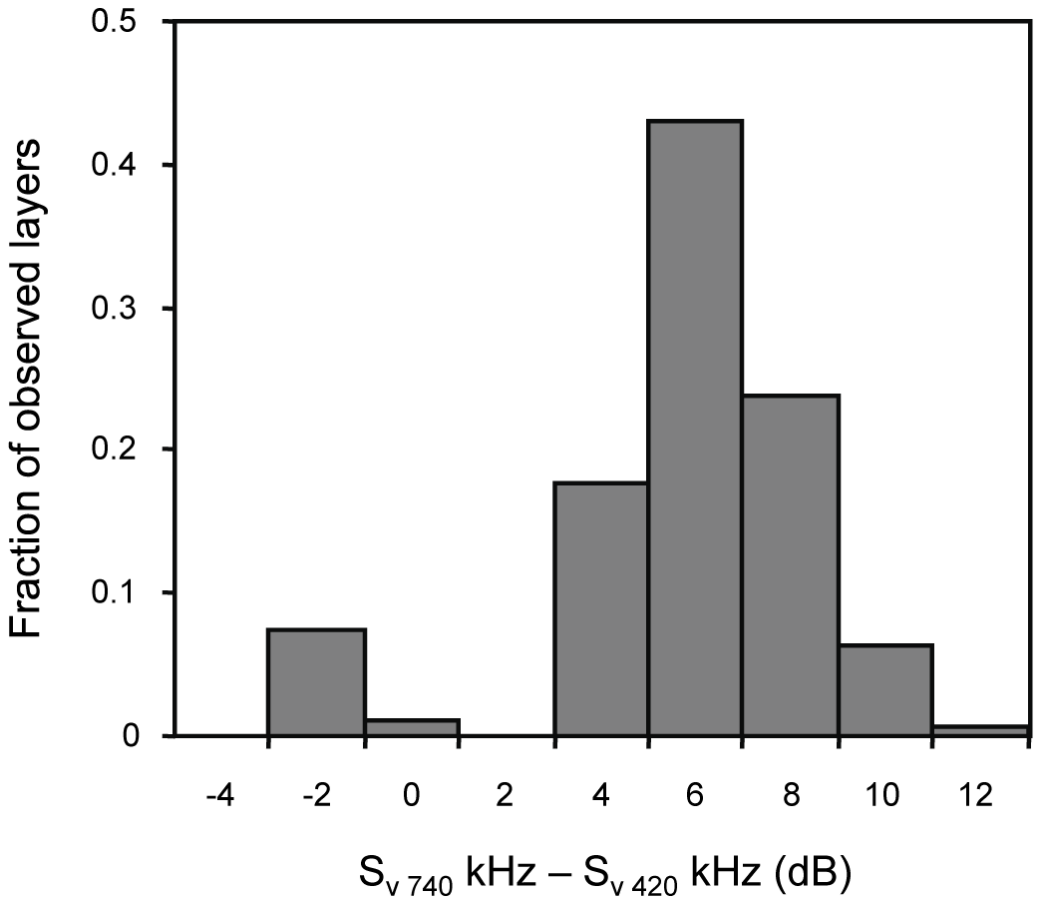
A histogram of the frequency response (S_v 740 kHz_ - S_v 420 kHz_) of layers detected by the moored echosounder. The y-axis shows the fraction of all observations accounted for by each 2 dB frequency response bin. There were no layers with frequency response values between −1.6 and 2.7 dB.

The frequency response (S_v 740 kHz_ - S_v 420 kHz_) of zooplankton layers detected by the mooring was bimodal with one mode centered at −2.5 dB and a larger mode at 5.5 dB (Figure 5). There were no layers with frequency response values between −1.6 and 2.7 dB. Layers with a positive frequency response were sampled using stratified net tows on 11 occasions. When these layers were found, net tows, both those that covered the entire water column and those that targeted acoustic features, were dominated in both number and biomass by calanoid copepods between 0.5 and 2.0 mm. The single most abundant group was *Clausocalanus* spp. A paired t-test showed that there was a significant increase in the density of copepods in net tows targeting the acoustic layers compared to those covering the entire water column (t = 86.92, df = 10, p>>0.05).

The smaller mode in frequency response from the moored echosounder was accounted for by 31 layers, all detected in the second half of the 2009 field experiment. On five occasions, layers with stronger scattering at 420 kHz relative to 740 kHz were discretely sampled. These net tows were unlike those taken at any other time during the experiment as they were dominated both in number and biomass by hyperiid amphipods with equivalent spherical diameters between 2.6–3.7 mm. Vertically integrated tows of the entire water column taken at the same time showed no difference from stratified tows in the total number of amphipods caught (paired t-test: t = 0.72, df = 4, p>>0.05). There was a significant increase in the total number of copepods in net tows covering the entire water column relative to the tows targeting just the layer (paired t-test: t = 11.86, df = 4 p<0.01) but no increase in copepod density (paired t-test: t = 1.20, df = 4, p>>0.05).

**Figure 6:**
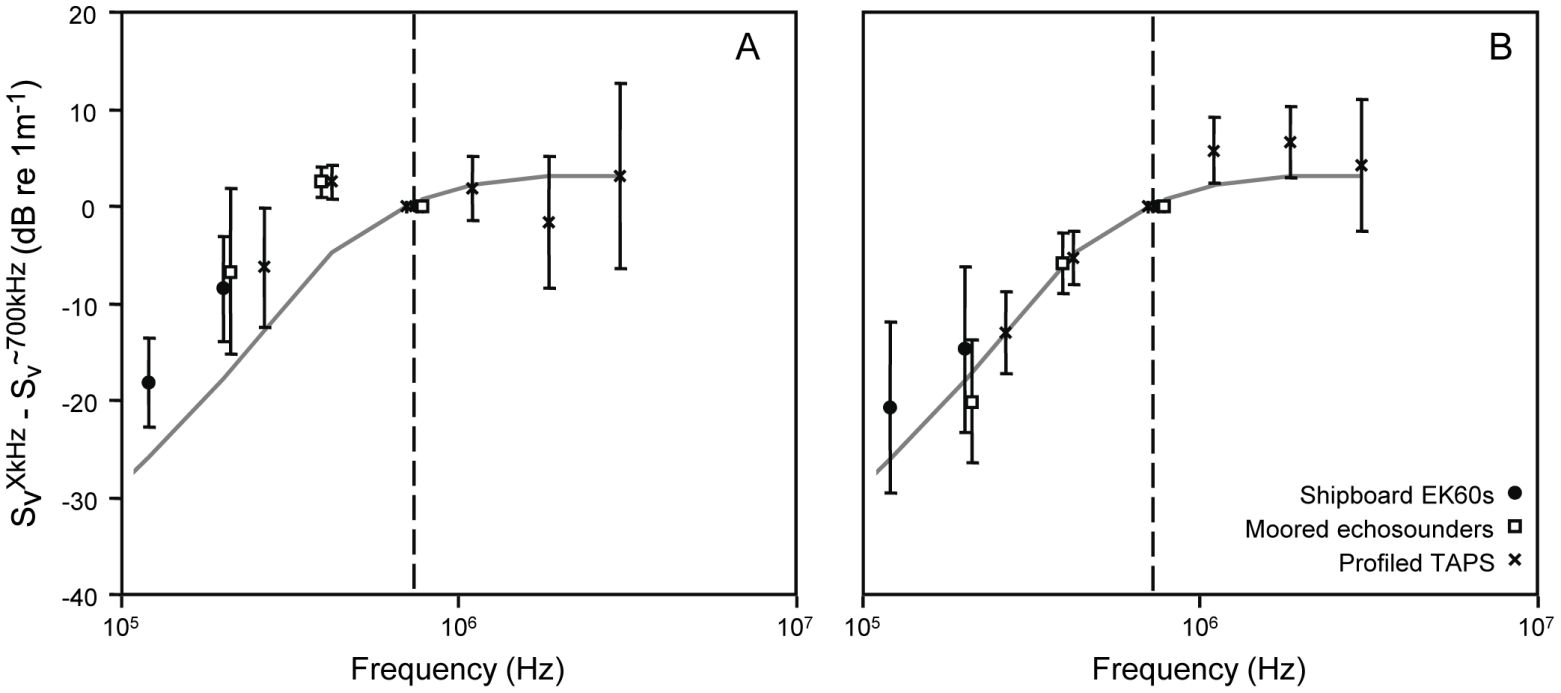
The mean volume scattering for each identified thin layer relative to that measured around 700(dashed line) is shown as a function of frequency for each of three acoustic instruments. Error bars show standard deviation. A) The frequency response of mode 1 layers, those that had stronger scattering at 420 kHz relative to 740 kHz in the moored data set. B) The frequency response of mode 2 layers. The gray line in each figure represents the scattering response expected for a fluid sphere of the median size of copepods measured from net tows.

During ship sampling, TAPS profiles were used to characterize the frequency response of scattering layers, which can provide information on the type and size of dominant scatterers. The frequency response of all layers was characteristic of small, fluid-like scatterers; generally increasing scattering strength with increasing frequency, sometimes reaching an asymptote at the highest frequencies with the presence of relatively small local nulls in scattering [41]. The average scattering responses for the two types of layers identified from the moored data are shown in Figure 6.

### Overlap of plankton layers

**Figure 7:**
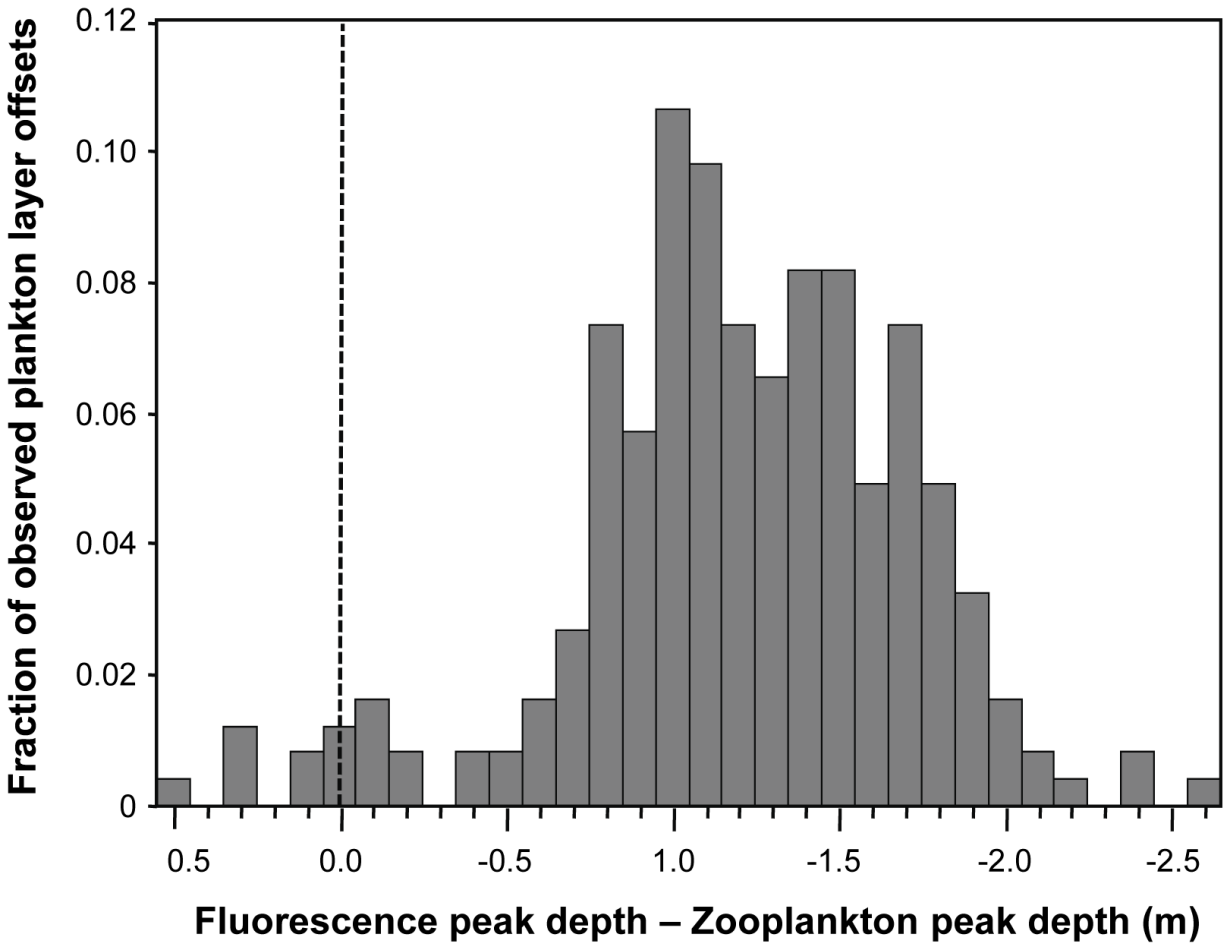
A histogram of the distance between the peaks of simultaneously detected fluorescence and zooplankton layers. The y-axis shows the fraction of all observations accounted for by data within each 0.1 m bin. Positive values indicate the zooplankton layer is shallower than the fluorescent feature while negative values mean the zooplankton layer is deeper in the water column than the fluorescent layer.

**Figure 8:**
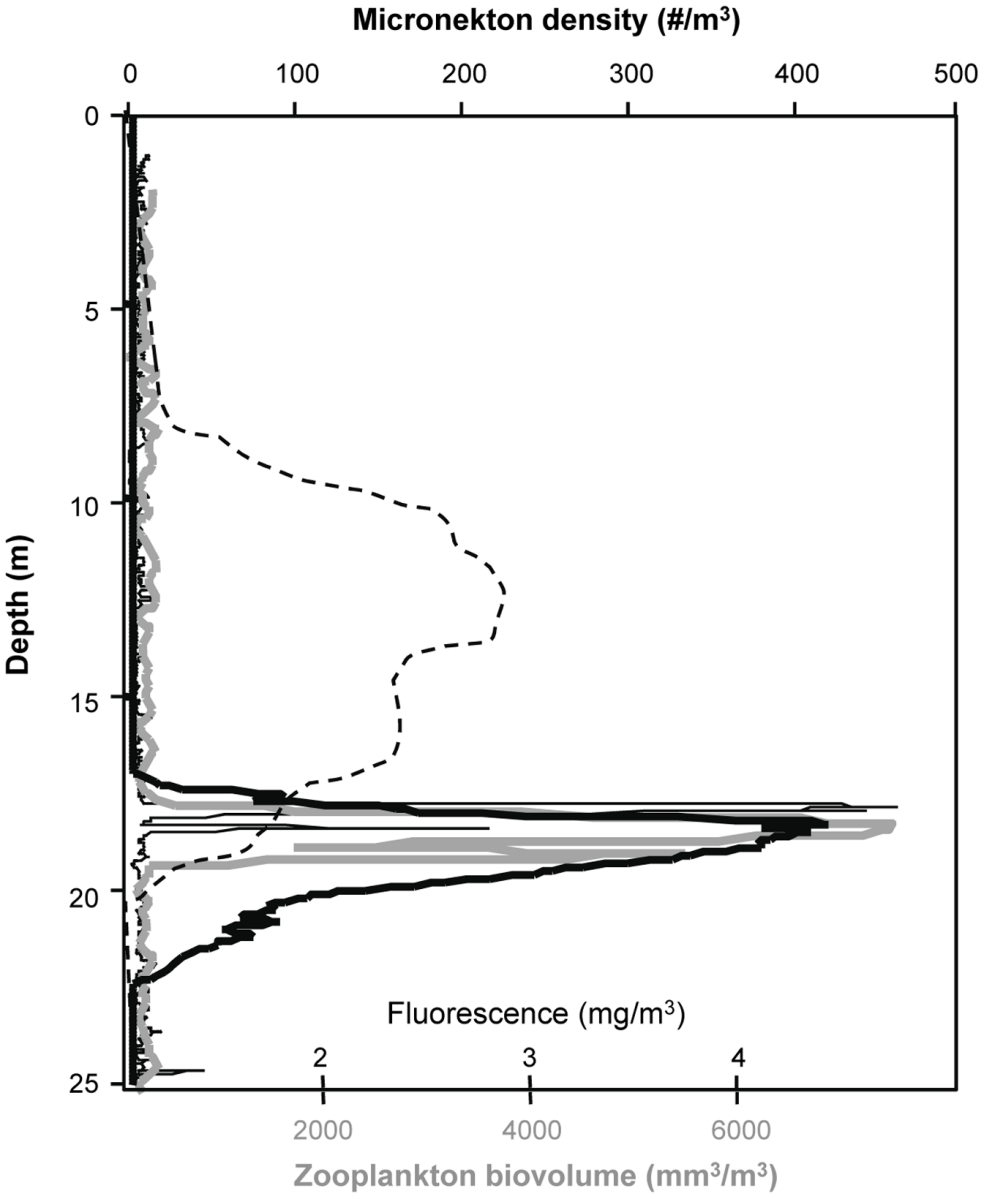
A sample profile of fluorescence (thin black line), zooplankton biovolume (gray line), and micronekton density (heavy black line) measured using shipboard sampling tools 30 minutes after sunset. The zooplankton layer shown was a mode 2 layer comprised of copepods. The vertical distribution of micronekton at the same time on the next night when no zooplankton layer was detected is shown as a dashed black line.

The absolute value of the offset between the peaks of co-occurring fluorescent and acoustically scattering layers is shown in Figure 3C. Despite the apparent parallel movements of fluorescent layers and zooplankton layers, the median offset between paired layer peaks was less than 2.5 m only between dusk and 0100 h. Given the average thickness of each layer type (∼2.5 m) and the fact that >90% of the layer's integrated fluorescence or acoustic scattering occurred within this thickness, this is the maximum separation that would allow consistent overlap between individuals within each layer type. Only at and immediately after dusk was the offset between layers consistently less than 2.5 m, with layers of zooplankton consistently deeper than phytoplankton layers (Figures 7 and 8).

### Zooplankton effects on micronekton and spinner dolphins

**Figure 9:**
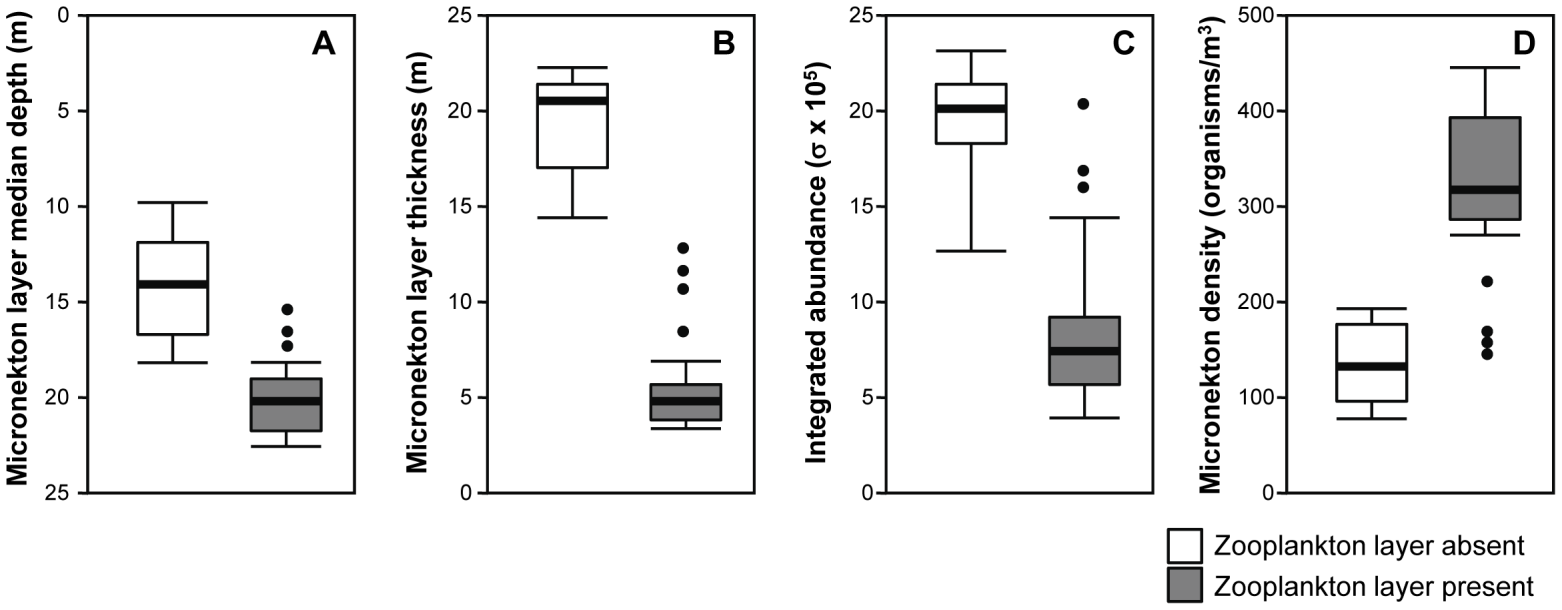
Micronekton layer characteristics 30 minutes after sunset when a zooplankton layer was absent (white boxes) and when a zooplankton layer was present (gray boxes). For all plots, the median of the distribution is shown by a heavy bar, the box represents one interquartile range, the error bars indicate the 95% confidence interval, and circles show outliers.

The diel migration of micronekton into the shallow waters of Oahu's shelf has been well described previously [31], [42]. The general patterns of micronekton during the time periods of this study matched those observations. For example, previous work has shown that nearly 100% of the micronekton found at night in these shallow waters were myctophids [31]. Both the camera system on the profiler and the frequency response of scattering from micronekton measured using the shipboard sampling indicated that held true during this study. However, while timing of arrival of the micronektonic layer at the 25 m site matched those observed previously and was not affected by layer presence (ANOVA: df = 1,25; F = 1.8; p = 0.41), the vertical location of the layer just after dusk showed more variation relative to previous studies that measured this time period with coarser temporal resolution [42]. The vertical distribution of micronekton at dusk on a night when a mode 2 zooplankton layer was detected is shown in Figure 8. The vertical distribution of micronekton at the same time on the next night when no zooplankton layer was detected is also shown. A multiple analysis of variance showed that the presence of zooplankton layers significantly affected the characteristics of micronekton layers measured just after dusk (df = 1,25; F = 68.3, p<0.001; Figure 9) but not later in the night (df = 1,25; F = 0.85, p = 0.91). Between subjects effects tests showed that there was a significant effect of layer presence on micronekton median depth (F = 24.3; p<0.005), micronekton layer thickness (F = 107.6; p<0.001), integrated abundance (F = 11.5; p<0.01), and micronekton density just after dusk (F = 46.9; p<0.001). A regression analysis showed a strong, linear relationship between the peak depth of the zooplankton layer and the peak depth of the micronekton layer observed on the same night, both 30 minutes after sunset (micronekton depth  =  0.96 * zooplankton depth – 1.05; r^2^ = 0.83; p<0.001).

The presence of zooplankton layers had a significant effect on the mean depth of dolphins observed in the 15–45 minutes after dusk (Mean dolphin depth: layer present = 19.6 m, absent = 12.3 m; ANOVA: df = 1,16; F = 17.13; p<0.005) but not during the 90–120 minutes after dusk (Mean dolphin depth = 10.28; ANOVA: df = 1,16, F = 1.7; p = 0.51). The presence of zooplankton layers had a significant effect on the number of groups of dolphins observed in the 15–45 minutes after dusk (Average number of groups: layers present = 4.8, absent = 2.6; ANOVA: F = 30.42; df = 1,16; p<0.001) but again, not during the later time period (Average number of groups = 3.8; ANOVA: F = 0.41; p = 0.88). The presence of layers but not sampling date significantly affected the measured characteristics of dolphin behavior just after dusk (MANOVA: df = 3,66; F_day_ = 0.13, p = 0.94; F_layers_ = 100.8, p<0.001) but neither was important later in the night (MANOVA: df = 3,66; F_day_ = 1.21, p = 0.71; F_layers_ = 1.42, p = 0.57). Between subjects effects tests for the time period just after dusk (df = 1,71) showed that there was a significant effect of layer presence on foraging dolphin group size (Mode group size: layers present = 20, absent = 24; F = 20.1; p<0.001) and average inter-pair spacing within the group (Average inter-pair spacing: layers present = 3.76 m, absent = 2.77 m; F = 261.1; p<0.001 for both comparisons) but not on time for a feeding event by an individual pair (mean = 10.2; F = 0.21, p = 0.65). Observations that involved the entire feeding stage of a foraging event (e.g., from the onset of pairs moving into the circle to surfacing) showed no significant effect of layer presence on the duration of the feeding stage of foraging events (df = 1,26; F = 1.07, p = 0.31). As a result, while the duration of each pair of dolphins' individual feeding event did not increase, the number of feeding events was affected by the presence of zooplankton layers, increasing from an average of 2.5 when layers were not present to 5.1 when layers were present.

### Micronekton effects on zooplankton layers

**Figure 10:**
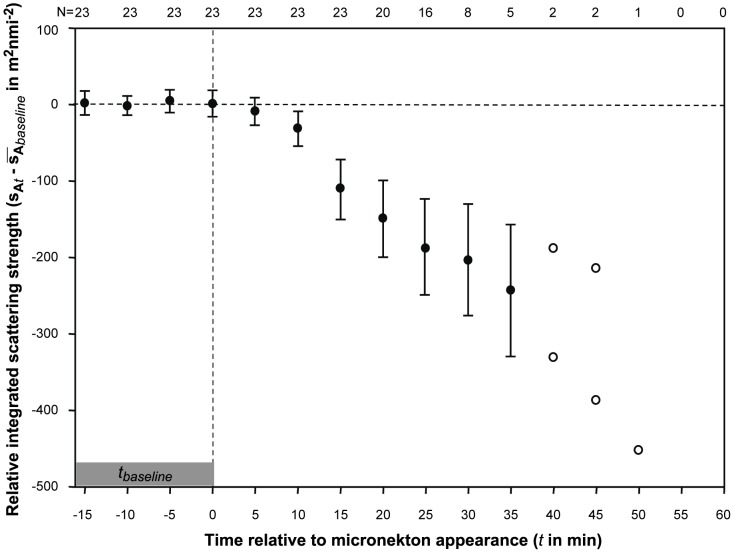
Changes in the biomass within zooplankton layers after the arrival of micronekton at the study site indicate rapid loss of these features each night. Scattering at 740-minute intervals before the arrival of micronekton. Mean changes relative to zero values are shown in 5 minute intervals (points) with error bars indicating 95% confidence intervals. When 2 or fewer layers were identified in a time interval, actual points are shown with open symbols. The number of layers in each time interval is indicated near the top of the plot.

To examine the effect of micronekton on zooplankton layers, integrated scattering at 740 kHz within a layer was normalized to the integrated scattering over the 15 minutes before the first detection of micronekton and tracked in 5-minute intervals from 15 minutes before to 60 minutes after micronekton arrived. The average value for each layer in the three, 5 minute intervals immediately before the arrival of micronekton was subtracted from each subsequent observation of the same feature to normalize the results. The integrated scattering strength began to decrease within 15 minutes and layers began to be lost entirely 25 minutes after micronekton arrived at the mooring station (Figure 10).

## Discussion

Our goal in this work was to add temporal dynamics to our growing understanding of spatial variance in ecosystem processes in Hawaii's nearshore pelagic environment. To do this, we used moored sensors to describe the fine-scale vertical dynamics of each component of system over a variety of timescales. By measuring true coherence between predators and prey in both space and time, we gain insight into the mechanisms underlying the interactions between these organisms. Considerable attention has been paid to micronekton aggregations [31], [42], [43], spinner dolphins [23], and more recently, phytoplankton aggregations [30], [44] at this site. Our results for these components of the system are individually consistent with previous descriptions. Micronekton are present in the nearshore only at night where primarily myctophid fishes achieve considerable densities. Spinner dolphins track the migratory patterns of micronekton, foraging primarily in cooperative groups that herd patches of micronekton into even more intense aggregations. The overall concentrations of phytoplankton in nearshore waters are relatively low, consistent with other subtropical habitats. However, the regular appearance of intense (up 5 mg m^−3^), thin (typically less than a few meters) layers dominated the fluorescence in the water column, accounting for up to 94% of the water column integrated value.

Zooplankton aggregations in this system have not been well characterized [29], [45] and the critical links involving zooplankton are only beginning to be explored [28]. During our two, 3-week study periods, zooplankton layers were relatively common, occurring during at least some time during every day of the study. During most of the study, net tows showed that zooplankton layers were made up of copepods, as was the remainder of the water column. These dense layers reflected the general composition of the entire system. However, during the second half of 2009, net tows within layers were dominated by hyperiid amphipods while in the remainder of the water column, copepods still dominated by number and biovolume. These differences in layer composition were reflected in differences in the frequency response of acoustic backscatter with features made up of copepods having higher scattering at 740 kHz while features dominated by hyperiid amphipods showed higher scattering at 420 kHz. The unique zooplankton layers found only in one week in the second half of the 2009 experiment occurred during a period of uncommonly persistent low wind speeds and increased stratification.

Zooplankton within layers dominated the zooplankton biomass measured in the water column when layers were present. Despite covering only an average of 10% of the water column's depth, zooplankton in layers accounted for between 31 and 100% of the measured zooplankton biomass. Over the course of our two, 3-week studies, the average concentrations of copepods in the water column were approximately 200 m^−3^ for a biovolume of 400 mm^2^ m^−3^. The average layer of zooplankton had a density of zooplankton at its peak of about 5,000 individuals m^−3^ while the strongest layer had a peak density of over 100,000 individuals m^−3^. However, these densities covered a very narrow vertical range, typically less than 3 m so the areal density of a layer was often lower than its peak density. Despite large differences in the peak intensity of layers and layer thickness, the integrated scattering within a layer was relatively constant at 250 m^2^ nmi^−2^ as thinner layers became more intense but did not contain more individual zooplankters. The origins of this biomass limit and its implications are not clear.

Zooplankton layers showed strong, predictable diel patterns in their occurrence and location in the water column. Zooplankton layers were most common during the afternoon, disappeared just after dusk, a pattern also observed in other sites in Hawaii [46], reappearing just before midnight. Zooplankton layers were found deep in the water column at night and an average of about 10 m shallower during the day. Around dusk, the abundant zooplankton layers showed a very narrow range of depths centered at 20 m, 5 m above the seafloor. This diel pattern is likely to be the result of behavioral dynamics of the zooplankters, perhaps as a response to convective overturning that occurs after the sun sets [30].

Layers of fluorescent phytoplankton also showed distinctive diel patterns with more layers observed at night than during the day with peaks in layer abundance at dawn and dusk. The peak in abundance at dusk is consistent with observations by McManus et al [30], whoalso observed a peak in abundance just before sunset. This peak is coincident with the time of day when thermal stratification is the strongest and most persistent [30], however, that stratification breaks down throughout the night due to convective overturn and thus cannot explain the somewhat smaller dawn peak in layer frequency. The depth of fluorescent layers of phytoplankton was restricted around dusk to just shallower than 20 m. Throughout the night, phytoplankton layers moved upwards through water column with an increasingly variable range of depths amongst them until they were within 7 m of the surface around dawn. During daylight hours, too few phytoplankton layers were identified to characterize their depth distribution. This limited number of detectable phytoplankton layers during the day could be due to dispersion of phytoplankton during the day followed by reformation into layers at dusk, migration of layers to the surface where they would not be detected by the moored or shipboard profilers as has been observed in layers of motile phytoplankton in other habitats [47], or perhaps intense non-photochemical quenching of fluorescence during the day.

The strong diel patterns of layers of both phytoplankton and zooplankton result in a highly variable potential for interaction between the two groups. Layers of phytoplankton and zooplankton show different temporal trends in their occurrence but similar patterns in their vertical position in the water column. However, despite the similar patterns in vertical position, layers that occurred at the same time did not often occur within a few meters of each other vertically and the zooplankton layer was always deeper than the phytoplankton layer so there was no potential for interaction during vertical crossings of layers. A significant number of phytoplankton and zooplankton within layers co-occur in both three-dimensional space and time at night, however, only just after dusk did the organisms in these layers predictably overlap. This is consistent with observations of other copepod species which focus their feeding during dusk and/or dawn [48]. However, the pattern was quite different from that observed in Monterey Bay, California where layers of zooplankton overlapped with phytoplankton infrequently but predictably as a function of fraction of available phytoplankton in layers and not diurnal cycling [40]. Interestingly, in the observations here, all phytoplankton layers contained at least 18% of the total phytoplankton fluorescence in the water column, the threshold at which zooplankton layers actively responded to phytoplankton layers in the richer, temperate waters of Monterey Bay, indicating different mechanisms for layer co-occurrence.

While not evidence of consumption, overlap between predator and prey is necessary for foraging and thus only during this less than one hour each day could zooplankton be consuming fluorescent phytoplankton found in layers in this system. However, despite the predictable co-location of layers of phytoplankton and zooplankton at dusk, complete overlap of these layers did not occur at this time or any other. The peak of zooplankton layers was always deeper than the peak of fluorescent layers. At the time of consistent co-location of layers, throughout all observations the peak of zooplankton layers was approximately 1 m deeper than the phytoplankton layer occurring at the same time. As a result, only the upper ∼40% of a layer of zooplankton interacts with the lower ∼30% of the phytoplankton layer. These offsets could be the result of physical forcing affecting these different sized organisms differently, a foraging tactic of the predator attacking prey from beneath, a response of consumers to their own predators, or a balance of potentially opposing forces. Offsets between layers of different types of plankton have been observed but only rarely have small offsets been predictable [40], [49], [50]. Benoit-Bird et al [40] found that when fluorescent and acoustic layers were in close proximity, layer peaks were not coincident but zooplankton were nearly equally likely to be above the phytoplankton layer as below it. So, while small offsets between layers of plankton appear to be common, the factors driving those relationships likely vary amongst systems and species.

Dusk is also a critical period for other interactions within the system. Shortly after sunset, micronektonic organisms, primarily myctophid fishes, ascend from the deep, aggregating on the narrow island shelf to consume the relatively rich resources present near the island at night [29], [31]. The phenology and typical vertical distributions of these organisms have been well documented [42]. We noticed dramatic departures from these typical patterns whenever dense layers of zooplankton were detected at dusk as illustrated in Figure 8. The upper edge of micronekton was not nearly as shallow as expected. This also affected the thickness of the layer as the bottom edge of the micronekton layer is constrained by the seafloor, resulting in an increase in the density of micronekton. The total abundance of micronekton within the layer, however, decreased as layers reached these high densities. The effects on micronekton were not observed to be as strong when zooplankton layers were made up of hyperiid amphipods as opposed to pelagic copepods. The outliers in Figure 9 were all measured from zooplankton layers with scattering at 420 kHz that exceeded that at 740 kHz, associated with large numbers of hyperiid amphipods in net tows through the layer. These outliers occurred in a single week during May of 2009, during a period of uncommonly persistent low wind speeds and increased stratification. A preference for copepods is consistent with the known diets of similar myctophid species in adjacent waters [51].

The presence of a dense, thin layer of zooplankton, particularly those made up of copepods, caused micronekton to abbreviate their vertical migration in these shelf waters. This change in behavior likely allows migrating micronekton to exploit the rich resource these layers represent. Net tows and acoustic sampling both reveal that each layer of zooplankton accounted for at least 30% of the total biomass of zooplankton in the water column, sometimes containing all of the water column's zooplankton biomass in the sampling location. It should not be surprising that zooplankton consumers that move vertically through such layers either stop moving when they reach the upper limit of these features or quickly move back into them, particularly if the original vertical movement was instigated as a quest for food as most assume nocturnal upward migration to be [52].

The effects of zooplankton layers on micronekton behavior were relatively short lived. Within an hour of their appearance, all micronekton were observed to resume their more typical vertical distribution. Within that same period of time, each zooplankton layer became undetectable as its integrated acoustic scattering gradually dropped (Figure 10). Layers of zooplankton began to disappear within 20 minutes of the arrival of midwater micronekton and all layers were undetectable after 50 minutes. The disappearance of these layers could be due to dispersion of individuals or to loss by consumption. After micronekton resumed their normal movement, layers of zooplankton reappeared beginning about 2 hours later. The reappearance of layers did not result in any measurable changes in micronekton despite their continued co-occurrence in the x-y plane and some vertical overlap between the lower edge of the micronekton layer and the zooplankton layer. The reappearance of zooplankton layers would suggest that dispersion plays some role in the disappearance of these features after dusk, however dramatically reduced peak intensities (e.g. 10x or more) indicate that consumption likely plays a role in their demise as well.

The short temporal overlap between micronekton and zooplankton layers might suggest a limited importance of these features in the energy acquisition of the micronekton that make a stop within them. However, simple calculations indicate that relative increases in foraging gains may indeed by significant. The density of zooplankton within these layers is at least two orders of magnitude higher than the average density of all zooplankton in the water column typical for the same time and location [29]. In fact, the biomass of zooplankton in the average layer exceeds by two-fold the average biomass of the entire water column in this site. If copepods are a preferred food source as indicated by the increased response to layers made up of copepods relative to those made up of amphipods, the relative local density difference is even more striking as zooplankton layers were not representative of the entire water column's zooplankton composition but rather an intensification only of the copepod community, increasing the ratio of preferred to non-preferred food resources which may increase foraging efficiency [53]. Combining estimates of biomass with Figure 10 shows that after about 30 minutes, the biomass of zooplankton in layers roughly equals the water column integrated average. If prey encounter rates for each myctophid are roughly related to the density of zooplankton in these two conditions, then those 30 minutes within the layer are equal to approximately 3.5 hours foraging under more typical conditions. If prey consumption is decreased proportionately with micronekton density, then the increased density of competitors during that time could reduce the benefit from a factor of 7 to a factor of ∼3, a likely underestimation of the benefit given results that indicate higher foraging success rates on more densely aggregated prey [54], [55], [56]. However, when foraging time is limited by the day night cycle to about half of each 24-period, even a factor of 3 for this short post-dusk time window would result in a ∼10% overall increase in foraging gains.

The presence of layers of zooplankton cascaded even further up the food, affecting the behavior of dolphins observed at dusk. On evenings when zooplankton layers were present, spinner dolphins were an average of 60% deeper in the water column, in groups that were 17% smaller, with spacing between pairs of dolphins 35% higher and were detected nearly twice as often relative to those evenings when a zooplankton layer was absent. Perhaps most importantly, the number of feeding events observed in each pair of dolphins and consequently the feeding time for each individual more than doubled when zooplankton layers were present. These changes only occurred in a 30 minute window just after dusk, the same time period where significant effects of layer presence were observed in micronekton. Some of the changes observed in dolphin behavior may involve costs (deeper diving), however the increase in the number of feeding events indicate a net gain as a result of zooplankton layers during the post-dusk time window. As spinner dolphin foraging is limited by feeding time rather than directly by the availability of food [23], the doubling in duration of feeding time over about 5% of their total foraging time each day could have significant impacts on an individual's fitness.

Previous analyses of data from the field efforts described here have shown the importance of the layers of organisms we observed in their interactions [28]. In part because of the intensity of these aggregations and strong, once per day patterns, correlations amongst trophic levels were robust to averaging over each day, a much longer time scale than the scale at which the interactions between organism types were occurring. However, this averaging obscured the details of these interactions. Here, we show that the interactions driving the overall patterns observed amongst trophic levels occur over the same very short time interval – within the hour following sunset each night. The pattern of spatial coincidence between predator and prey is an emergent outcome of a behavioral race between them [11], [57]. In this case, the outcome we observed is the result of a number of interdependent races. The solutions for each step in the trophic level converged at a short, dynamic period of each day – dusk. Given the changes in physical processes [30] and the remarkable redistribution of the animals in the ecosystem [29], [42] that occurs at this time, foraging and avoidance tradeoffs change quickly during this time, ultimately allowing a brief period of intense interaction between species. It has long been recognized that dawn and dusk “transition periods” are dynamic and often critical periods for a variety of species [58], [59], [60], [61] and, as a result, for predator prey interactions [4]. However, dawn and dusk have often been explicitly excluded from analyses of diurnal patterns because of the challenges of obtaining sufficient samples to describe these short periods.

To understand the interactions between predators and prey in pelagic systems, we need concurrent data from each that is coincident and time and space. However, even under ideal conditions, we must recognize that studies that provide only snapshots in time are likely to miss the critical periods when interactions occur amongst species, leaving us with weak or inconsistent correlations between predator and prey, a common observation in pelagic ecosystems [5], [6], [62]. One approach that has proven helpful in dealing with this challenge for single predator-prey pairs is to utilize the identification of foraging behavior to focus sampling efforts [10]. Multi-trophic level interactions, however, present additional challenges. Because of the importance of place in Hawaii's slope associated ecosystem, a fixed network of sensors that could sample with relatively fine resolution in vertical space over time allowed us to identify a critical time window for predator-prey coherence throughout the food chain. Long-term, multifaceted arrays of ocean sensors are or will soon be coming online in a variety of pelagic systems. We are optimistic that these observational tools will provide opportunities to move beyond examination of temporally separated snapshots towards mechanistic descriptions of ecosystems. Space use decisions by predator and prey determine the spatial overlap between the two that affects encounter rates, predation rates and population and community dynamics [63]. These space use decisions are dynamic over a variety of time scales, particularly in the constantly moving fluid environment of the pelagic. While few studies of spatial ecology have explicitly included a temporal component at scales smaller than seasonal or larger than single behavioral interactions or simple day/night contrasts [22], our observations that short time windows can drive the structure and function of a complex suite of organisms highlight the importance of explicitly adding a temporal component to our descriptions of heterogeneity.
